# Impact of Maternal Feed Restriction at Different Stages of Gestation on the Proteomic Profile of the Newborn Skeletal Muscle

**DOI:** 10.3390/ani12081011

**Published:** 2022-04-13

**Authors:** Thaís Correia Costa, Luana Lucas Dutra, Tiago Antônio de Oliveira Mendes, Marta Maria dos Santos, Renata Veroneze, Mateus Pies Gionbelli, Marcio de Souza Duarte

**Affiliations:** 1 Department of Animal Science, Universidade Federal de Viçosa, Viçosa 36570-000, Brazil; thais.correia@ufv.br (T.C.C.); marta.fontes@ufv.br (M.M.d.S.); renata.veroneze@ufv.br (R.V.); 2 Muscle Biology and Nutrigenomics Laboratory, Universidade Federal de Viçosa, Viçosa 36570-000, Brazil; 3 Department of Biochemistry and Molecular Biology, Universidade Federal de Viçosa, Viçosa 36570-000, Brazil; luana.dutra@ufv.br (L.L.D.); tiagoaomendes@ufv.br (T.A.d.O.M.); 4 Department of Animal Science, Universidade Federal de Lavras, Lavras 37200-900, Brazil; mateus.pg@ufla.br; 5 Department of Animal Biosciences, University of Guelph, Guelph, ON N1G 2W1, Canada

**Keywords:** *Capra hircus*, energy metabolism, feed restriction, maternal nutrition, proteome, skeletal muscle

## Abstract

**Simple Summary:**

Due to forage seasonality, pregnant ruminants raised in pasture are commonly subjected to a period of feed restriction, which may lead to changes in the skeletal muscle metabolism of the offspring. Such alteration may have long-term consequences for the offspring’s performance and carcass composition. In the current study, we evaluated the effects of feed restriction at different stages of gestation on the proteomic profile in the skeletal muscle of the offspring. The results showed that both periods of restriction, whether applied in the first or last half of gestation, influence muscle energy metabolism of the progeny. Specifically, the restriction in the first half of gestation influences muscle glycogen utilization, fatty acid oxidation, and the energy-investment phase of glycolysis, while the restriction in the second half of gestation influences the energy-generation phase of glycolysis and the production of glutamine in the skeletal muscle of the offspring.

**Abstract:**

We aimed to investigate the effects of the maternal plane of nutrition during gestation on the proteome profile of the skeletal muscle of the newborn. Pregnant goats were assigned to the following experimental treatments: restriction maintenance (RM) where pregnant dams were fed at 50% of their maintenance requirements from 8–84 days of gestation, and then feed of 100% of the maintenance requirements was supplied from 85—parturition (*n* = 6); maintenance restriction (MR) where pregnant dams were fed at 100% of their maintenance requirements from 8–84 days of gestation, and then experienced feed restriction of 50% of the maintenance requirements from 85—parturition (*n* = 8). At birth, newborns were euthanized and samples of the *Longissimus dorsi* muscle were collected and used to perform HPLC-MS/MS analysis. The network analyses were performed to identify the biological processes and KEGG pathways of the proteins identified as differentially abundant protein and were deemed significant when the adjusted *p*-value (FDR) < 0.05. Our results suggest that treatment RM affects the energy metabolism of newborns’ skeletal muscle by changing the energy-investment phase of glycolysis, in addition to utilizing glycogen as a carbon source. Moreover, the RM plane of nutrition may contribute to fatty acid oxidation and increases in the cytosolic α-KG and mitochondrial NADH levels in the skeletal muscle of the newborn. On the other hand, treatment MR likely affects the energy-generation phase of glycolysis, contributing to the accumulation of mitochondrial α-KG and the biosynthesis of glutamine.

## 1. Introduction

Improvement in the efficiency of livestock production has been largely demanded due to population growth, in addition to cost reduction, food safety, and quality. Among the cost reduction approaches, feed restriction is commonly adopted postnatally as a strategy of compensatory growth, which consists in the application of a period of undernutrition, followed by realimentation, to promote the increase in the animal’s growth rate and directly reduce costs of animal feed [1]. However, feed restriction during certain periods of gestation may negatively impact the embryo and fetus development, leading to long-term consequences that may impact postnatal growth and health [2,3]. Specifically, the early gestational period in utero development is markedly characterized by fetal organ development, as well as the beginning of skeletal muscle development where primary and secondary myogenesis occurs, while the majority of intramuscular adipogenesis and fibrogenesis occur in the last half of gestation [4]. Moreover, it is well-established that muscle hyperplasia and most of the fibro-adipogenic progenitor cells are limited to the prenatal period [4]. Hence, depending on the period in which maternal feed restriction is applied, distinct biological processes may be activated or deactivated by the action of transcripts, proteins, or metabolites, modifying the whole course of cell fate in the skeletal muscle of the progeny.

Commonly, pregnant ruminants raised in pasture are subjected to a period of feed restriction, resulting from the lack of quantity and quality of forages. Hence, under this situation, maternal metabolism prioritizes the delivery of nutrients for the formation of vital tissues instead of secondary tissues, such as skeletal muscle, which is a disadvantage in animals raised for meat production [5,6]. Studies evaluating the impacts of maternal undernutrition on offspring’s development, health, and performance have been reported, using targeted approaches [7] as well as sequencing-based approaches for profiling mRNAs (RNAseq) [3,8]. Although RNAseq provides important information about differentially expressed genes related to biological events comparing different experimental scenarios, the expression of mRNAs does not reflect its translation in a functional protein [9], as the mRNA transcription is only partially associated with the abundance of proteins [10]. Therefore, proteomic approaches have been demonstrated to be a useful tool in livestock studies that provides the identification of differentially abundant functional proteins, allowing the establishment of its interaction and enriched molecular pathways [11].

Given the importance of meat production for human consumption, the mechanisms involved in skeletal muscle development have been widely studied over the past years [12]. Muscle proteins are classified based on their function and solubility and can be divided into sarcoplasmic, myofibrillar, and structural proteins [13]. The sarcoplasmic fraction plays roles in regulating cellular metabolism, where the glycolytic enzymes comprise the majority of sarcoplasmic proteins [14]. Because the utilization of substrate for producing energy for skeletal muscle development is dependent on maternal nutritional status, we hypothesized that maternal feed restriction programs the skeletal muscle proteome of the offspring, based on alterations of sarcoplasmic proteins. Moreover, fractioning the protein extract allows the identification of proteins of interest that are often less expressed and hard to detect [15,16]. Besides the expansive potential of proteomics, studies linking the effects of maternal nutrition at different stages of gestation with alteration of the offspring’s skeletal muscle proteome are still little explored in livestock animals. Thus, in the present study, we aimed to investigate the impact of the maternal plane of nutrition where pregnant dams were feed-restricted at different stages of gestation (first or second half) on the proteomic profile of the skeletal muscle of newborns.

## 2. Materials and Methods

### 2.1. Animals and Sampling

The Ethical Committee on Animal Use of the Department of Animal Science at Universidade Federal de Viçosa, Minas Gerais, Brazil (protocol number 09/2017) approved all the procedures prior to the beginning of the experiment.

The detailed description of the experimental management practices was previously described in [17]. Briefly, 14 nulliparous pregnant dairy goats were randomly assigned to one of the following dietary treatments: treatment RM (*n* = 6) consisted of feed at 50% of maintenance requirement from 8–84 days of gestation followed by feed at 100% of maintenance requirement from day 85 of gestation to parturition (~150 days), while the treatment MR (*n* = 8) consisted of animals fed at 100% of maintenance requirement from 8–84 days of gestation and then fed at 50% of maintenance requirement from day 85 of gestation to parturition. It is worth mentioning that effects of maternal nutrient restriction compared to feed adhering to the nutrient requirements result in the impairment of the offspring’s skeletal muscle development [2,8,18,19]. Thus, the choice of the experimental design was based on the aim of evaluating what metabolic processes were likely affected by varying the maternal plane of nutrition when the restriction occurs during the gestational period.

The dams were weighed once a week and the week of gestation was taken into consideration for the adjustment of the dry matter intake, in order to meet the objective of the experimental treatments. The same diet was offered to the dams for both treatments and consisted of 111.6 g/kg of crude protein (CP) and 676 g/kg of total digestible nutrients (TDN) on a dry matter (DM) basis, which was composed of corn silage (723 g/kg DM basis), soybean meal (96 g/kg DM basis), ground corn (165 g/kg DM basis), and mineral mixture (16 g/kg DM basis). The amount of dry matter offered to the dams adhered to the nutritional requirements for pregnant dairy goats [20], meeting either 50% or 100% of their requirements according to the experimental treatments.

After birth, male newborn goats were immediately separated from the dams, stunned using a non-penetrating captive bolt pistol, and exsanguinated. In the case of twins (4 in RM and 5 in MR), the heaviest newborn goat was selected to be sampled. Skeletal muscle samples (~1 g) were collected from the *Longissimus* muscle and stored in liquid nitrogen for further protein extraction.

### 2.2. Protein Extraction

Sarcoplasmic protein fraction of *Longissimus* muscle was extracted from 0.1 g of tissue in 1 mL lysis buffer (20 mM Tris HCl pH 8, 5 mM EDTA, 1% 2-mercaptoethanol, and 1% protease inhibitor cocktail (Sigma-Aldrich^®®^, San Luis, MO, USA), homogenized using a shaft-type homogenizer (LabGEN 125, Cole-Parmer, Bunker Hill, IL, USA). The homogenate was centrifuged at 20,200× *g* for 20 min at 4 °C. The supernatant was collected, aliquoted, and stored at −80 °C. Sarcoplasmic protein content was estimated by Bradford Protein Assay (Bio-Rad, Hercules, CA, USA).

### 2.3. Protein Identification and Data Processing

Proteins were digested using a solution containing 50 mM ammonium bicarbonate and 20 ug trypsin (Promega, Madison, WI, USA) overnight at 37 °C. Tryptic peptides were then dried using a SpeedVac centrifuge (AG-22331, Eppendorf, Hamburg, Germany), resuspended with trifluoroacetic acid (TFA), and desalted using Zip-Tip, according to the manufacturer’s protocol. Samples were again dried in SpeedVac centrifuge (AG-22331, Eppendorf, Germany) and sent to Central Analítica of IQ-USP (São Paulo, Brazil) for protein identification and quantification performed in a NanoAquity high-performance liquid chromatographer (HPLC) coupled with a maXis 3G high-resolution Q-TOF mass spectrometer (Bruker Daltonics, Bremen, Germany). The raw data were processed with MaxQuant (v. 1.6.3.3) software with parameters set to default values, considering the protein amino terminal acetylation, methionine oxidation as variable modification, and the fixed modification as carbamidomethylation of cysteine. The trypsin specificity was kept as the digestion mode and the instrument selected was Bruker-QTOF, set to default, including the parameters of first (20 ppm) and main (10 ppm) search peptide tolerance. For the calculation of the label-free quantification (LFQ) protein intensity, the LFQ mode was added and at least two unique peptide ratios (min LFQ ratio count = 2) were considered. The goat reference proteome used was obtained from UniProt database (ID: UP000291000) available in (www.uniprot.org) (accessed on 27 September 2019).

### 2.4. Network Analyses

The Kyoto Encyclopedia of Genes and Genomes (KEGG) pathways enrichment analysis was built with String version 11.0 (string-db.org) (accessed on 1 January 2020). The interaction network of the exclusive proteins from the treatments MR and RM was obtained using the available interaction map from the closest species (*Ovis aries*), with the default option (medium confidence given by score of 0.4) and connections defined according to statistical evidence. KEGG pathways were considered significantly enriched based on adjusted FDR [21] using *p*-value < 0.05. For the differentially abundant proteins (DAPs), the network analysis was built in ClueGo 2.5.3 [22], a Cytoscape 3.7.2 application. The biological processes shared by the DAPs were identified based on a unilateral hypergeometric test also adjusted by FDR [23,24]. Only the biological processes identified with the adjusted *p*-value < 0.05 were considered for posterior analysis involving protein networks. In this context, the node colors represent the functional group; whereas the node size represents the term enrichment significance, being the additional inferences on edges connection based on the Kappa statistic (Kappa score = 0.4) [25,26].

### 2.5. Statistical Analysis

Statistical analysis was performed based on a peptide-based model through the MSqRob software (version 0.7.6) [27,28]. The peptide intensities were log2-transformed and normalized based on the robust linear regression (RLR) method. Prior to analysis, proteins identified by site, reverse, and potential contaminants were filtered and removed. The statistical model considered the fixed effects of treatments (RM and MR), the fixed effect of the number of offspring (1 or 2), and the random effect of sequence. The random effect was included to provide fixed effects estimation independently of the peptide effect. Since we aimed to investigate the treatment effects, the contrasts were made between treatments comparing RM vs. MR, with significance based on ANOVA and posterior differentially abundant proteins (DAPs) adopting *p*-value (FDR) < 0.05.

## 3. Results

### 3.1. Differentially Abundant Proteins

**Figure 1:**
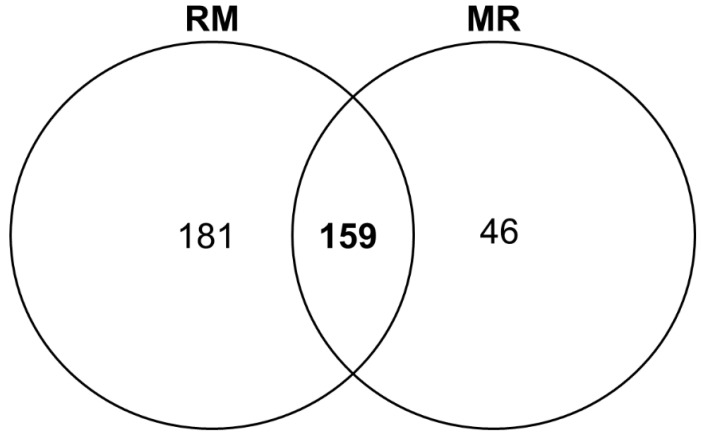
Venn diagram of the proteins identified. Number of proteins identified in each treatment (exclusive), the intercept containing the number of proteins common in both treatments. RM = restriction maintenance; MR = maintenance restriction.

Initially, a total of 415 proteins were identified (Table S1) in the skeletal muscle of newborn goats. However, after removing the proteins only identified by site, potential contaminants, and reverse sequences, a total of 386 proteins were maintained. We found 181 and 46 exclusive proteins in the treatments RM and MR, respectively (Figure 1). For DAPs, 159 proteins corresponding to common proteins present in both treatments (intercept) were tested (Figure 1).

**Figure 2:**
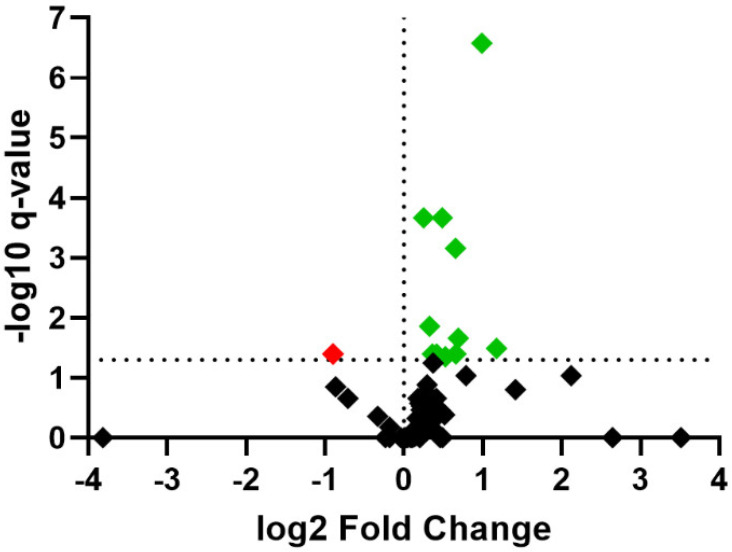
Volcano plot comparing protein abundance fold changes (FC) between treatments (ratio RM/MR). Differentially abundant proteins (DAPs; FDR < 0.05) are highlighted in red and green. DAPs highlighted in green are upregulated and DAPs highlighted in red are downregulated. The black squares represent the non-significant proteins (FDR > 0.05).

Controlling the *p*-value adjusted (FDR) < 0.05, a total of 13 proteins were differentially abundant between treatments (Figure 2, Table 1). Among them, ATP-dependent 6-phosphofructokinase (PFKM), dihydrolipoyl dehydrogenase (DLD), lipoyl-binding domain-containing protein (DLST), S-formylglutathione hydrolase (ESD), IF rod domain-containing protein (DES), triosephosphate isomerase (TPI1), and 5 uncharacterized proteins corresponding to the genes *FLNC*, *HSPA9*, *SELEMBP1*, and *MYOM1* were more abundant in RM compared with MR (FRD < 0.05; Table 1). Calponin homology (CH) domain-containing protein (SMTNL1) and pyruvate kinase (PKM) were less abundant in RM compared to MR (FDR < 0.05; Table 1).

### 3.2. Protein-Protein Interaction Network and KEGG Pathways of the Exclusive Proteins

The interaction network of the exclusive proteins from treatment RM was significant (*p*-value (FDR) < 1 c 10^−16^), while in the treatment MR, the *p*-value was higher than RM (*p*-value (FDR) < 1.8 × 10^−5^); however, it was still significant, indicating that the proteins are at least partially biologically connected.

KEGG analysis from the exclusive proteins of treatment RM indicate pathways of interest related to tight junction, carbon metabolism, valine, leucine, and isoleucine degradation, mRNA surveillance pathway, fatty acid degradation, RNA transport, long-term potentiation, tryptophan metabolism, regulation of actin cytoskeleton, and citrate cycle (TCA cycle) (Table 2).

Although the proteins exclusive to MR treatment showed fewer interactions between them, interesting pathways were enriched, such as glycolysis/gluconeogenesis, citrate cycle (TCA cycle), biosynthesis of amino acids, carbon metabolism, metabolic pathways, HIF-1 signaling pathway, pyruvate metabolism, arginine and proline metabolism, and phagosome and 2-oxocarboxylic acid metabolism (Table 2).

### 3.3. Interaction Network and Functional Enrichment of Differentially Abundant Proteins

**Figure 3:**
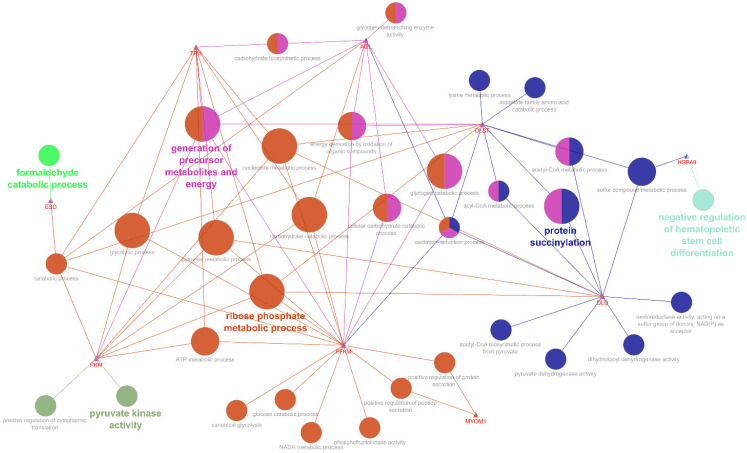
Analyses of the enriched biological process from the differentially abundant proteins (DAPs). The triangles represent the protein names, while the circles represent the biological processes. Different node color means distinct functional group, and node size means the significance. The most significant (FDR < 0.05) term is labeled with bold letters.

Due to the presence of uncharacterized proteins identified between the DAPs, we built a network based on gene names. Moreover, all DAPs (more or less abundant proteins) were combined into the same network. The DAPs network revealed enriched biological processes and sub-biological processes related to negative regulation of hematopoietic stem cell differentiation (HSP9A), protein succinylation (DLD, DLST), ribose phosphate metabolic process (PFKM, PKM, TFI1, DLD, DLST), generation of precursor metabolites and energy (AGL, TFL1, PKM, PFKM), formaldehyde catabolic process (ESD), pyruvate kinase activity (PKM), and various sub-processes of interest, such as, positive regulation of protein secretion (MYOM1), glycolytic process (TFL1, PFKM, PKM), and lysine metabolic process (DLST), among others (Figure 3).

## 4. Discussion

Through the differences previously reported for the average daily gain of maternal tissues between treatments [17], the effectiveness and the objective of the experimental treatments in causing feed restriction at different time-points of gestation were reached. As such, we were successfully able to assess the consequences of the plane of nutrition where restriction occurred only in the first or last half of gestation for the proteome profile of the newborn skeletal muscle.

Maternal feed restriction impairs the offspring’s skeletal muscle development, causing long-lasting impact throughout the animals’ productive life. The mechanisms of placenta adaptation in the face of a scarce intrauterine environment ensure fetus survival, and may cause permanent structural and functional changes by programming its metabolism through changes in transcripts, proteins, and metabolite profile [29]. Given the importance of vital tissues for fetuses (i.e., organs and viscera), the lack of nutrients during gestation prioritizes nutrient delivery for this purpose, while skeletal muscle development may be impaired. Thus, with the employment of proteomic approaches in the current study, we have identified the differentially abundant proteins (DAPs) and exclusively expressed proteins in the skeletal muscle of the offspring, resulting from maternal feed restriction during the first or last half of gestation. Specifically, the current study identified 13 DAPs involved in general biological processes mostly related to muscle energy metabolism. From the exclusive expressed proteins (181 in RM and 46 in MR), we performed a network analysis and the identification of KEGG pathways revealed enriched pathways related to fatty acid degradation, glycolysis/gluconeogenesis, citrate cycle (TCA cycle), and biosynthesis of amino acids, among others. Although the enriched biological processes and pathways were similar between treatments, the protein abundance inherent to each treatment affects different steps of the pathway and thus influences the muscle energy balance.

The skeletal muscle characteristics of plasticity allow its metabolism to change and adapt according to the environment, such as calorie and nutrient intake [30]. In terms of metabolic properties, skeletal muscle is the primary site of glucose uptake and storage [31]. Glucose is oxidized to generate ATP through two major pathways: the oxidative (aerobic) and the glycolytic (anaerobic) pathway. Through the aerobic pathway, glucose undergoes glycolysis to generate pyruvate, followed by its conversion into acetyl-coA, which enters the citrate cycle, producing substrates for the electron transport chain which results in ATP synthesis.

**Figure 4:**
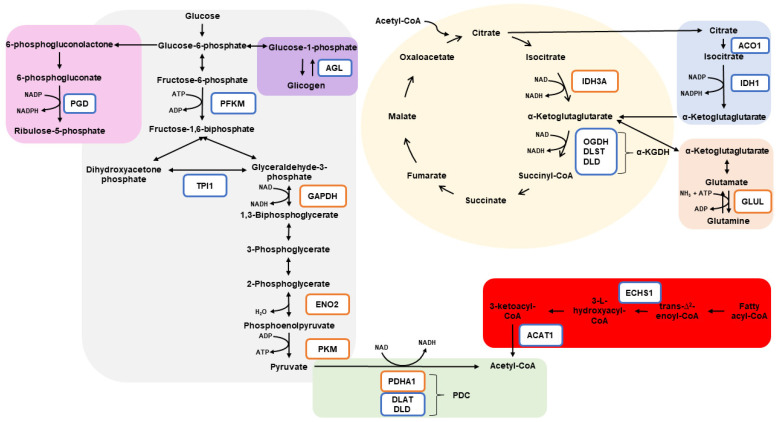
Summarized metabolic pathways influenced by the experimental treatments. Glycolysis is represented in gray, glycogenolysis in purple, pentose phosphate pathway in pink, oxidative decarboxylation of pyruvate to form acetyl-CoA in green, citrate cycle in yellow, cytoplasmic conversion of citrate into α-ketoglutarate in blue, glutamine synthesis in orange, and fatty acid degradation in red. Proteins inside orange squares were exclusively expressed in MR or were more abundant in MR compared to RM. Proteins inside blue squares were exclusively expressed in RM or were more abundant in RM compared to MR. α-KGDH: α- ketoglutarate dehydrogenase complex; ACAT1: acetoacetyl-CoA thiolase; ACO1: aconitase 1; AGL: glycogen debranching enzyme; DLAT: dihydrolipoyl transacetylase; DLD: dihydrolipoamide dehydrogenase; DLST: dihydrolipoyllysine-residue succinyltransferase; ECHS1: enoyl CoA hydratase, short chain 1; ENO2: enolase 2; GAPDH: glyceraldehyde-3-phosphate dehydrogenase; GLUL: glutamine synthetase; IDH1: isocitrate dehydrogenase 1; IDH3A: isocitrate dehydrogenase 3 catalytic subunit alpha; OGDH: 2-oxoglutarate dehydrogenase; PDC: pyruvate dehydrogenase complex; PDHA1: pyruvate dehydrogenase E1 subunit alpha 1; PFKM: phosphofructokinase; PGD: 6-phosphogluconate dehydrogenase; PKM: pyruvate kinase; TPI1: triosephosphate isomerase.

The proteins found to be differentially abundant and exclusive in our experimental treatments appear in biological processes (BP) and signaling pathways (SP) associated with energy metabolism, such as glycolytic process (BP), pyruvate metabolism (SP), glycolysis/gluconeogenesis (SP), and citrate cycle (TCA cycle; SP) (Figure 4).

### 4.1. Glycolysis

Glycolysis is commonly divided into the energy-investment and energy-generation phases [32,33,34]. Among the more abundant proteins in RM compared to MR, PFKM and TPI1 play roles in the energy-investment phase of glycolysis. The enzyme PFKM uses ATP to catalyze the irreversible conversion of fructose-6-phosphate into fructose-1,6-bisphosphate (FBP). FBP is then cleaved into glyceraldehyde 3-phosphate (GAP) and dihydroxyacetone phosphate (DHAP). DHAP is a precursor of lipid synthesis, while GAP is utilized in the next steps of glycolysis. Moreover, DHAP and GAP can be reversibly converted by the reaction involving the enzyme TPI1 [35]. A recent study showed that DHAP may function by signaling the availability of glucose to activate the complex of proteins mTORC1 [36], which is an important regulator of cellular growth, protein synthesis, autophagy, and lipogenesis [37]. Studies evaluating post-mortem glycolysis and meat quality parameters observed that TPI1 abundance was associated with beef tenderness [38,39] and was positively related to the rate of pH decline, which may partially be associated with meat color [40,41,42,43]. Therefore, since TPI1 may influence the abundance of both metabolites (DHAP and GAP), and consequently, the synthesis of lipids and ATP, the greater abundance of TPI1 in the skeletal muscle of animals born from dams that were feed-restricted in the first half of gestation compared to those born from dams feed-restricted in the last half of gestation may not be attributed to a specific pathway (lipid or ATP synthesis). However, we speculate that the greater abundance of TPI1 in the skeletal muscle of offspring from RM treatment would negatively impact the conversion from muscle into meat upon slaughter later in life, with consequences for meat color and tenderness parameters.

According to Akram [32], when glycolysis is initiated from the glycogen breakdown (glycogenolysis), fewer ATPs are consumed in the first phase of glycolysis, because in this case, the first step of glucose activation is dispensable. In this context, the protein AGL, which is an important protein in the process of glycogenolysis, was more abundant in the treatment RM compared to MR. Such an observation may indicate that, despite the ATP utilization in the reaction catalyzed by the PFKM, the skeletal muscle of the offspring from treatment RM may use glycogen as its main carbon source, and consequently, more ATP would be spared from the first step of glycolysis.

The second phase of glycolysis, named the energy-generation phase, is characterized by the oxidative conversion of GAP to pyruvate and the formation of ATP and NADH [34]. Three enzymes that participate in this phase of glycolysis were exclusively expressed in the treatment MR or were less abundant in RM compared to MR. GAPDH, which was exclusively detected in treatment MR, catalyzes the reversible conversion of GAP into 1,3-biphosphoglycerate, in addition to generating NADH from this reaction [32]. An important glycolytic enzyme, ENO2, was also exclusively expressed in the treatment MR. Although ENO2 (gamma-enolase) is one of the enolase isoforms found to be predominantly located in the neuron and neuroendocrine tissues [44], ENO2 was found to be expressed in human and rat cultured muscle cells [45]. The reversible reaction that converts 2-phosphoglycerate into the energy-rich phosphoenolpyruvate (PEP) is catalyzed by ENO2 [32,46]. After that, PEP is dephosphorylated in a reaction catalyzed by the enzyme PKM, downregulated in RM compared to MR, leading to the final glycolytic step where pyruvate and ATP are formed [46]. Thus, the results related to glycolysis indicate that the treatment RM may cause an enhancement in the energy-investment phase of glycolysis, while treatment MR may have a major function favoring the energy-generation phase of glycolysis.

Once formed, pyruvate may follow different pathways. In our current study, we identified proteins in both treatments that participate in the same destiny as pyruvate. The oxidative decarboxylation of pyruvate to form acetyl-CoA, CO2, and NADH is catalyzed by a well-orchestrated complex called pyruvate dehydrogenase complex (PDC). The PDC is composed of three catalytic subunits: pyruvate dehydrogenase (PDH), dihydrolipoamide acetyltransferase (DLAT), and dihydrolipoamide dehydrogenase (DLD) [47,48]. Intriguingly, PDHA1, which is a component of the PDH subunit, was exclusively expressed in the treatment MR. On the other hand, the proteins that are components of the subunit DLAT were exclusively expressed in treatment RM, while proteins that are components of DLD were more abundant in RM compared to the MR group. Because our experimental treatment was based on feed restriction, it is possible that some vitamins may have been differentially metabolized by the dams, and consequently, their availability to the fetuses was different between treatments. For instance, vitamin B1 is a cofactor of the subunit PDH [49], while vitamin B5 is the key precursor for the biosynthesis of CoA [49], utilized as a cofactor in subunit DLAT, and the precursors of FAD and NAD are vitamin B2 [49] and B3 [50], respectively, which serve as cofactors in the subunit DLD.

### 4.2. Citrate Cycle (TCA)

Citrate cycle (TCA) initiates catabolizing of acetyl-CoA molecules through the steps involving many enzymes that produce reducing equivalents NADH and FADH, which will be further driven to ATP production in the electron-transport chain [46]. The first step of TCA produces citrate, and like pyruvate, citrate may follow a variety of pathways both inside and outside the mitochondria matrix, in addition to regulating intermediate metabolites. When cellular iron levels are high, the enzyme ACO1, which was found to be exclusively expressed in treatment RM, catalyzes the reversible conversion of the cytosolic citrate into isocitrate [51,52]. Cytosolic isocitrate is then metabolized by the enzyme IDH1, also found to be exclusively expressed in treatment MR, generating α-ketoglutarate (a-KG) and NADPH [52], which is an important cofactor involved in lipid and cholesterol metabolism [53,54]. Moreover, findings from Moreno et al. [55] showed that ACO1 was positively associated with adipogenic markers, linking the iron metabolism with the adipogenic potential of the adipose tissue. Thus, our results may suggest that, compared to treatment MR, maternal feed restriction during the first half of gestation may influence the iron metabolism in the skeletal muscle of the offspring, which may result in enhancement of the protein abundance of ACO1 and IDH1 and likely increase the biosynthesis of lipids.

Treatment RM may also influence the pathways downstream a-KG, since the components of the a-KG dehydrogenase (α-KGDH) complex were found to be exclusively expressed in treatment RM (OGDH) or were more abundant in RM compared to MR (DLAT and DLST). The a-KGDH complex function catalyzed the conversion of a-ketoglutarate to succinyl-CoA and produced NADH [56]. Thus, based on these results, RM treatment, not only influences the a-KG production in cytosol but also its downstream reaction on the mitochondria, leading to the enhancement of NADH synthesis.

### 4.3. Glutamine

Regarding the cytosolic formation of α-KG, in contrast to our findings in treatment RM, treatment MR may have influenced the increase in mitochondrial α-KG, since the enzyme responsible for the conversion of the mitochondrial isocitrate into α-KG (IDH3A) was exclusively expressed in treatment MR. The amount of α-KG in mitochondria is dependent on the state of oxidation reduction (redox). For example, high levels of NAD over NADH lead to the conversion of α-KG into succinyl-CoA, while high levels of NADH over NAD lead to α-KG accumulation and consequently its participation in several other biological processes, such as the biosynthesis of amino acids. To prevent the excess of α-KG in the cells, reactions associated with the production of glutamine may occur [57]. The protein GLUL, exclusively expressed in the treatment MR, uses ammonia to catalyze the conversion of glutamate into glutamine [58,59]. When required, skeletal muscle releases glutamine into the blood, supplying the energy and protein requirements of other cells and tissues [60]. Moreover, glutamine oxidation is crucial for energy production and survival of pluripotent stem cells, in addition to donating nitrogen in de novo nucleotide synthesis [61]. Taken together, the exclusive expression of the proteins IDH3A and GLUL in the skeletal muscle of the offspring from treatment MR may indicate the accumulation of mitochondrial α-KG and its fate of glutamine synthesis, increasing the availability of this amino acid, which can be further utilized in several biological processes.

### 4.4. Fatty Acid Degradation

Under the aerobic conditions, ATP production is obtained through three key biological pathways, including the citrate cycle and fatty acid degradation. As previously discussed, our experimental treatments may have affected the overall skeletal muscle energy metabolism. The proteins ACAT1 and ECHS1 found to be exclusively expressed in treatment RM are involved in the fatty acid beta-oxidation signaling pathways. ECHS1 catalyzes the second reaction, while ACAT1 catalyzes the last reaction, ending in acetyl-CoA synthesis. Our results were contrary to the study which reported the downregulation of the corresponding genes in bulls subjected to compensatory growth conditions (restriction followed by realimentation) [62], reinforcing that the results may depend on the period of feed restriction (prenatal vs. postnatal) or post-transcriptional factors. Interestingly, ACAT1 can acetylate the lysine residues of PDHA1, causing inhibition of the PDC in some conditions and favoring cell proliferation [63]. Hence, the exclusive expression of PDHA1 in treatment MR in particular may be related to the exclusive expression of ACAT1 in RM, which is able to inhibit this protein. Taken together, these findings may result in increased fatty acid degradation in the skeletal muscle of the offspring from treatment RM.

### 4.5. Pentose Phosphate Pathways

Interestingly, the nucleotide metabolism may have been affected by our experimental treatment. The protein PGD was exclusively expressed in treatment RM, and it links the cytosolic carbohydrate metabolism with protein secretion [64]. PGD is a NADP-dependent enzyme that catalyzes the decarboxylation of 6-phosphogluconate to ribulose 5-phosphate (Ru5P), producing NADPH in the aerobic stage of the pentose phosphate pathways [46]. NADPH is mainly used in the processes of cellular redox balance and antioxidant defense [65], while the Ru5P is used for the synthesis of nucleotides and nucleic acids [46]. Therefore, the greater production of NADPH generated by the reaction involving PGD may be required in the skeletal muscle of the offspring, resulting from maternal feed restriction at the first half of gestation.

## 5. Conclusions

In conclusion, our data indicate that the maternal plane of nutrition where feed restriction occurs at different stages of gestation affects the overall proteins related to energy metabolism in the skeletal muscle of the newborn. Specifically, our results showed that maternal feed restriction during the first half of gestation followed by non-restriction regulates proteins related to the energy-investment phase of glycolysis. On the other hand, the energy-generation phase of glycolysis is steeper in the skeletal muscle of the offspring resulting from dams that were not feed-restricted at the first half of gestation and were feed-restricted at the second half of gestation. Moreover, carbon sources may be largely provided by glycogen in the skeletal muscle of the offspring born from dams feed-restricted at the first half of gestation. Indeed, skeletal muscle of newborns from dams feed-restricted at the first half of gestation had an increase in cytosolic α-KG; however, it appears that α-KG is used to produce NADH in mitochondria. On the other hand, in the newborns from dams feed-restricted at the second half of gestation, the α-KG accumulation in mitochondria appears to have deviated for the biosynthesis of glutamine. Furthermore, proteins involved in fatty acid degradation were enhanced by feed restriction at the first half of gestation, in addition to influencing the NADPH production by regulating an enzyme that participates in the pentose phosphate pathways. However, it is possible that offspring from a dam undergoing feed restriction during the first half of gestation likely experience extensive use of glycogen and fatty acid storage. Although the long-term effects of maternal nutrition on skeletal muscle development and metabolism have been exhibited in previous studies, if the changes observed in our trial persist in the skeletal muscle throughout the progeny’s life, they need to be further investigated.

## Figures and Tables

**Table 1:** Differentially abundant proteins (DAPs) in the skeletal muscle of the offspring.

Accession	Protein Name	Gene Name	FDR ^1^	Fold Change ^2^
				(RM vs. MR)
A0A452DPE6	Calponin homology (CH) domain-containing protein	SMTNL1	0.0398564	−0.90
A0A452ET82	Pyruvate kinase	PKM	0.0398564	−0.90
A0A452FHP7	Uncharacterized protein	FLNC	0.0002162	0.25
A0A452FBM2	Uncharacterized protein	HSPA9	0.0138994	0.33
A0A452G4K3	ATP-dependent 6-phosphofructokinase	PFKM	0.0398564	0.36
A0A452FWD9; A0A452FWC6	Dihydrolipoyl dehydrogenase	DLD	0.0398564	0.42
A0A452FX48I; A0A452FWP3	Uncharacterized protein	AGL	0.0002162	0.49
A0A452ESM1; A0A452ERW8; A0A452ERN3	Lipoyl-binding domain-containing protein	DLST	0.0446152	0.53
A0A452EYA9; A0A452EYB9; A0A452EY59	Uncharacterized protein	SELENBP1	0.0006971	0.66
A0A452FIG7	S-formylglutathione hydrolase	ESD	0.0398564	0.66
A0A452EJS4	Uncharacterized protein	MYOM1	0.0219753	0.69
A0A452DWL1	IF rod domain-containing protein	DES	0.0000003	0.99
A0A452ET55	Triosephosphate isomerase	TPI1	0.0321358	1.18

RM = restriction maintenance; MR = maintenance restriction; ^1^ FDR= false discovery rate; ^2^ negative and positive fold change indicates the less and more abundant proteins in the treatment RM compared to MR.

**Table 2:** Enriched metabolic pathways of the exclusive proteins from treatment RM and MR.

KEGG ID	Description	FDR ^1^	Protein Names ^2^
Treatment RM			
oas04530	Tight junction	9.44 × 10^−8^	**ACTN1**, **ACTN4**, **EZR**, **LOC443340**, **MSN**, **MYH1**, **MYH13**, **MYH4**, **MYH8**, **MYL6**, **OMYHC2A**, PPP2R1A, **PPP2R1B**, **RDX**, **RHOA**, **YWHAQ**
oas01200	Carbon metabolism	7.73 × 10^−7^	**ACAT1**, **ACO1**, **ADH5**, **DLAT**, **ECHS1**, HADHA, **IDH1**, **OGDH**, **OGDHL**, **PGD**
oas00280	Valine, leucine, and isoleucine degradation	2.98 × 10^−5^	**ACAT1**, **ALDH2**, **ECHS1**, HADHA, **HIBADH**, HSD17B10, OXCT1
oas03015	mRNA surveillance pathway	5.52 × 10^−5^	**EIF4A3**, **PABPC1**, **PABPC4**, **PPP1CA**, **PPP1CB**, **PPP1CC**, PPP2R1A, **PPP2R1B**
oas00071	Fatty acid degradation	6.03 × 10^−5^	**ACAT1**, **ADH5**, **ALDH2**, **ECHS1**, ECI1, HADHA
oas03013	RNA transport	6.03 × 10^−5^	**EEF1A2**, **EIF4A1**, **EIF4A2**, **EIF4A3**, EIF4EBP1, **PABPC1**, **PABPC4**, SEC13, SUMO2, SUMO3
oas04720	Long-term potentiation	6.03 × 10^−5^	**PPP1CA**, **PPP1CB**, **PPP1CC**, PPP1R1A, PPP3CA, PPP3CB, PPP3CC
oas00380	Tryptophan metabolism	9.75 × 10^−5^	**ACAT1**, **ALDH2**, **ECHS1**, HADHA, **OGDH**, **OGDHL**
oas04810	Regulation of actin cytoskeleton	1.30 × 10^−4^	**ACTN1**, **ACTN4**, **EZR**, **LOC443340**, **MSN**, **PPP1CA**, **PPP1CB**, **PPP1CC**, **RDX**, **RHOA**
oas00020	Citrate cycle (TCA cycle)	1.80 × 10^−4^	**ACO1**, **DLAT**, **IDH1**, **OGDH**, **OGDHL**
Treatment MR			
oas00010	Glycolysis/Gluconeogenesis	2.68 × 10^−5^	**AKR1A1**, **ALDH7A1**, **ENO2**, **PDHA1**, **PDHA2**, **GAPDH**
oas00020	Citrate cycle (TCA cycle)	2.68 × 10^−5^	**IDH3A**, IDH3G, **PDHA1**, **PDHA2**
oas01230	Biosynthesis of amino acids	2.68 × 10^−5^	ARG1, **ENO2**, **GLUL**, **IDH3A**, IDH3G, **GAPDH**
oas01200	Carbon metabolism	9.08 × 10^−5^	**ENO2**, **IDH3A**, IDH3G, **PDHA1**, **PDHA2**, **GAPDH**
oas01100	Metabolic pathways	0.00064	**AKR1A1**, **ALDH7A1**, ARG1, **ENO2**, **GLUL**, **IDH3A**, IDH3G, LAP3, MTHFD1, PDHA1, **PDHA2**, **GAPDH**
oas04066	HIF-1 signaling pathway	0.00074	EIF4E, **ENO2**, **PDHA1**, **PDHA2**, **GAPDH**
oas00620	Pyruvate metabolism	0.0012	**ALDH7A1**, **PDHA1**, **PDHA2**
oas00330	Arginine and proline metabolism	0.0019	**ALDH7A1**, ARG1, **LAP3**
oas04145	Phagosome	0.0033	**RAC1**, **TUBB**, **TUBB1**, **TUBB2A**
oas01210	2-Oxocarboxylic acid metabolism	0.0055	**IDH3A**, IDH3G

RM = restriction maintenance; MR = maintenance restriction; ^1^ FDR= false discovery rate; ^2^ bold font are the proteins that are present in each treatment that are connected into the protein-protein interaction network.

## Data Availability

The raw data are provided in the supplementary table. Any additional data will be made available upon reasonable request to the corresponding author.

## Supplementary Materials

The following supporting information can be downloaded at: https://www.mdpi.com/article/10.3390/ani12081011/s1, Table S1: Sheet containing all the proteins identified, its respective molecular weight, and the LFQ intensities of each replicate.
